# Computer genomics research at the bioinformatics conference series in Novosibirsk

**DOI:** 10.1186/s12864-019-5846-3

**Published:** 2019-07-11

**Authors:** Yuriy L. Orlov, Elvira R. Galieva, Alexander V. Melerzanov

**Affiliations:** 1 Digital Health Institute, I.M. Sechenov First Moscow State Medical University of the Ministry of Health of the Russian Federation (Sechenov University), 119991 Moscow, Russia; 2 Novosibirsk State University, 630090 Novosibirsk, Russia; 3 Moscow Institute of Physics and Technology (MIPT), 141700, Dolgoprudny, Moscow Region, Russia

In this issue we present original research articles and highlight the studies on computational genomics discussed at BGRS\SB (Bioinformatics of Genome Regulation and Structure\Systems Biology) multi-conference (http://conf.bionet.nsc.ru/bgrssb2018/en/). The meetings on bioinformatics and systems biology BGRS\SB in Novosibirsk have been held biannualy since 1998. The conference committee prepared series of special post-conference journal issues at range of journals covering computational genomics and biomedicine applications since 2014 (https://bmcgenomics.biomedcentral.com/articles/supplements/volume-15-supplement-12). Recent special issues of BioMed Central journals after the conference include manuscripts on genomics, bioinformatics and genetics, and collated as *BMC Genomics* (https://bmcgenomics.biomedcentral.com/articles/supplements/volume-20-supplement-3) [1]*, BMC Medical Genomics* [2], *BMC Systems Biology* [3], *BMC Bioinformatics* [4], *BMC Evolutionary Biology* [5], *BMC Genetics* [6], *BMC Medical Genetics* [7], and *BMC Plant Biology* [8] Supplements this year.

The main idea of the research discussed at BGRS meetings might be formulated as regulation - regulation of gene expression at transcription level, regulation of protein functions at chromosome level, regulation of physiological function of cell, regulation of gene function at organism level [9]. System biology approaches for studying of cell function regulation could be described by the theory of gene networks - the science area presented at first BGRS-1998 [9, 10]. Previous BGRS conference 2016 had been presented at *BMC Genomics* (https://bmcgenomics.biomedcentral.com/articles/supplements/volume-17-supplement-14) [11] and BioMed Central journals as well [12–14]. Fast development of high-throughput sequencing technologies extended range of applications lead to creation of more complex dynamical models [15, 16] and enhancement of databases based on genome-wide studies [17].

To review bioinformatics meetings in Novosibirsk let us note «Belyaev Readings-2017» - memorial conference on genetics devoted to 100th anniversary of the birth of Academician Dmitri K. Belyaev (1917–1985), an outstanding scientist, evolutionist and geneticist (http://conf.bionet.nsc.ru/belyaev100/en). The series of BioMed Central journals in 2017 highlighted bioinformatics research from the Belyaev memorial conference [18–23] accompanied by “Vavilov journal of genetics and breeding” special journal issue [24]. The BGRS\SB-2018 conference got highlights at *Frontiers in Genetics* thematic issue “Bioinformatics of Genome Regulation and Systems Biology” with the title resembling abbreviation of BGRS\SB conference name (https://www.frontiersin.org/research-topics/8383/bioinformatics-of-genome-regulation-and-systems-biology).

Here we selected the articles on computational molecular biology and biomedicine applications discussed at BGRS-2018 multi-conference [25, 26]. Below is a summary of these papers and the article in *BMC Molecular and Cell Biology* [27] and *BMC Medical Genomics* [28] parallel issues with relevant references and background work.

Evgeniya Omelina and co-authors [25] present here the study on optimization of PCR conditions for Massively Parallel Reporter Assay (MPRA). This approach enables high-throughput functional evaluation of various DNA regulatory elements and their mutant variants. The assays are based on construction of highly diverse plasmid libraries containing two variable fragments, a region of interest and a barcode separated by a constant spacer sequence. The authors identified PCR parameters that ensure synthesis of specific (non-chimeric) products from highly diverse MPRA plasmid libraries.

This application is accompanied by the original research paper by Gera Pavlova et al. [27] from the same science group describing Patronin gene function in mitosis at *BMC Molecular and Cell Biology* special issue. Patronin belongs to the conserved protein family CAMSAP (calmodulin-regulated spectrin-associated proteins), which members bind the polymerizing microtubule minus ends. The precise role of Patronin in mitotic spindle assembly is poorly understood. G.A. Pavlova and colleagues show that Patronin associates with different types of microtubule bundles within the *Drosophila* mitotic spindle, and that it is required for their stability.

Liudmila Smirnova and co-authors [26] used omics technologies to study proteomes in schizophrenia and bipolar disorder. Since diagnostics of mental disorders is based only on clinical symptoms, there is a necessity in development of additional methods of diagnostics. The search for blood based biomarkers is important for modern biomedicine. Comparison of proteome profiles of different patient groups revealed sets of proteins specific for schizophrenia and bipolar disorder.

Elena Ignatieva et al. [28] present exome-wide search and functional annotation of genes associated in patients with severe tick-borne encephalitis. The paper published is at separate *BMC Medical Genomics* issue “Selected articles from BGRS\SB-2018: medical genomics (part 2)”. The authors identified set of candidate genes harboring rare, potentially pathogenic variants in exomes of patients with encephalitis and analyzed their function, protein-protein interactions. Whole-exome sequencing followed by systems biology approach enabled to identify eight candidate genes (MAP4, WDFY4, ACTRT2, KLHL25, MAP2K3, MBD1, OR10J1, and OR2T34) that can potentially determine predisposition to severe forms of tick-borne encephalitis.

To highlight genomics studies at presented at first BGRS-2018 special issue of *BMC Genomics* this year (https://bmcgenomics.biomedcentral.com/articles/supplements/volume-20-supplement-3) note the work by Wang and coauthors [29] discussing method for chromosome structure analysis based on integration of DNA sequences, ChIP-Seq and ChIA-PET data. The authors proposed the HidPET (Hierarchical and Dynamics Analysis of TF Cooperation with ChIA-PET and ChIP-Seq Data) continuing work on 3D genome modeling presented first at BGRS-2014 conference [30].

A.A.Yurchenko et al. [31] at same *BMC Genomics* issue published work on animal genomics - high-density genotyping on sheep breeds. The authors performed a high-density genotyping and comprehensive scans for signatures of selection in the genomes from 15 local sheep, found candidate genes related to morphology, adaptation, and domestication as well as wool quality and related features.

D.Konina and colleagues [32] presented study LINC01420 - long noncoding RNA. It was shown that some lncRNAs have small open reading frames (smORFs) that produce the functional microproteins. Konina and co-authors investi analyzed gated widely expressed lncRNA LINC01420, the function of which was not described at the time of the beginning of the study. D’Lima et al. [33] found smORF in the first exon of the LINC01420 gene. This smORF produces functional microprotein called non-annotated P-body dissociating polypeptide (NoBody) [34]. Konina and colleagues provided new facts about LINC01420 and its function.

L.A.Fedoseeva et al. [35] discussed the differences in brain stem transcriptional profiling in rat models of hypertension. The authors revealed set of differentially expressed genes and discussed their association with hypertension and blood pressure regulation, considered association with central nervous system diseases. This study continues research on transcriptome profiling on selection rats models [36] at the Institute of Cytology and Genetics SB RAS including published in *BMC Genomics* special issues in 2016 [37].

Concluding genomics research review at BGRS\SB-2018 conference note article by N.V.Ivanisenko et al. [38], work in plant genomics by I.V.Rozanova and coauthors [39].

Another important bioinformatics meeting in Novosibirsk - International Young Scientists School “Systems Biology and Bioinformatics” (SBB) (http://conf.bionet.nsc.ru/bgrssb2018/en/school/), an educational School on systems biology has own history and publications record in BMC thematic issues [40–42], see also *BMC Genomics* Supplement (https://bmcgenomics.biomedcentral.com/articles/supplements/volume-16-supplement-13). Note SBB-2019 event to be held in Novosibirsk in June 2019 (http://conf.bionet.nsc.ru/sbb2019/en/) in parallel to Plantgen-2019 conference. These meetings continue traditions of bioinformatics conference series in Novosibirsk organized by the Institute of Cytology and Genetics SB RAS and Novosibirsk State University. The great traditions of bioinformatics education summer schools to be continued this year in Russia by the event organized by Moscow Institute of Physics and Technology (MPTI) and Bioinformatics Institute in St. Petersburg (https://bioinf.me/education/summer/2019).

Concluding this short review of bioinformatics research post-conference thematic issues on genomics we invite our readers worldwide to participate next educational bioinformatics meetings - Systems Biology and Bioinformatics Young Scientists School SBB-2019 in Novosibirsk, Forum on Digital Medicine at Sechenov University and Bioinformatics School in Moscow in 2019. The most significant materials discussed at these meetings will be presented at new *BMC Genomics* Supplement in 2019. Next BGRS\SB-2020 multiconference is scheduled for summer 2020 in Novosibirsk, Russia.

## Data Availability

Not applicable.
